# A Study of the Antioxidant, Cytotoxic Activity and Adsorption Properties of Karelian Shungite by Physicochemical Methods

**DOI:** 10.3390/antiox10071121

**Published:** 2021-07-14

**Authors:** Liubov Skrypnik, Olga Babich, Stanislav Sukhikh, Olga Shishko, Svetlana Ivanova, Oleg Mozhei, Ivan Kochish, Ilia Nikonov

**Affiliations:** 1 Institute of Living Systems, Immanuel Kant Baltic Federal University, A. Nevskogo Street 14, 236016 Kaliningrad, Russia; LSkrypnik@kantiana.ru (L.S.); olich.43@mail.ru (O.B.); stas-asp@mail.ru (S.S.); Olga.shishko88@mail.ru (O.S.); imozhei1@kantiana.ru (O.M.); 2 Natural Nutraceutical Biotesting Laboratory, Kemerovo State University, Krasnaya Street 6, 650043 Kemerovo, Russia; 3 Department of General Mathematics and Informatics, Kemerovo State University, Krasnaya Street 6, 650043 Kemerovo, Russia; 4 Department of Zoological Hygiene and Poultry Breeding Named after A.K. Danilova, Moscow State Academy of Veterinary Medicine and Biotechnology Named after K.I. Skryabin, Academician Skryabin Street 23, 109472 Moscow, Russia; Kochish.i@mail.ru (I.K.); ilnikonov@yandex.ru (I.N.)

**Keywords:** shungite, antioxidant activity, amperometric method, spectrophotometric method

## Abstract

This study reveals that fossil shungite samples exhibit antioxidant activity, can reduce oxidized components, and bind to free radicals. A sample of Sh20 (size fraction—20 μm) (1.30 mg equivalents of ascorbic acid/g of shungite; 3.46 mg equivalents of trolox/g of shungite; 0.99 mg equivalents of quercetin/g of shungite) had the maximal activity according to the amperometric method. The obtained data indicate that shungite has antioxidant properties, but these are approximately 1000 times less pronounced than those of quercetin. A ShT20 sample (size fraction—20 μm + heat treatment) was found to have the highest antioxidant activity against the 2,2-diphenyl-1-picrylhydrazyl radical and cytotoxicity. Further studies, including the optimization of the antioxidant extraction conditions of shungite, and the analysis of the qualitative and quantitative composition of the obtained extracts, are required for a more accurate interpretation of the results. Shungite can be applied as an alternative to activated carbon in water purification, due to its absorption, catalytic, antioxidant, regenerating, and antibacterial properties, as well as its high environmental safety and relatively low cost. It is possible to identify new structural forms of carbon within, and other valuable properties of, shungite substance, which will make it possible to create effective technologies for the practical use of shungite rocks, particularly in the production of fullerenes and other carbon nanoclusters.

## 1. Introduction

Shungite, with its unique origin and massive reserves, has no analogs in the geological evolution of the Earth. Shungite is a part of the Paleoproterozoic carbon strata of Karelia, usually found in Zaonezhie, Russian Karelia, and forms 25 × 10^10^ tons of native organic matter over a large area [1,2]. 

The mineral form of shungite is a non-graphitizable fullerene-like carbon, which differs from graphite in its supramolecular, atomic, and ribbon (electronic) structure [3]. A distinctive feature of shungite is its ability to form a spherical structure (an empty sphere). At the atomic level, in addition to hexagonal rings consisting only of graphite, there are also pentagonal and heptagonal rings with a fullerene-like structure. At the level of the layered structure, the total excitation energy of the π- and σ-electrons of the valence electrons of the outer and inner levels is lower than in graphite, which is also a characteristic feature of fullerenes. Shungite ore from some sediments exhibits the diamagnetic properties of fullerenes. The texture of shungite is similar to the texture of glassy crystalline materials, where highly dispersed crystals are distributed in an amorphous matrix [4].

Research of shungites has been ongoing for over 200 years [5]. Depending on the carbon content, it is customary to distinguish five types of shungite rocks; the most common are minerals with a 30 wt% carbon concentration [6]. 

Since ancient times, shungite has been used to treat allergies, skin diseases, diabetes mellitus, stomatitis, periodontal disease, hair loss, cosmetic imperfections, and many other diseases [7]. It is characterized by high reactivity at elevated temperatures, significant adsorption capacity, catalytic activity, and the ability to conduct electric current. By the end of the twentieth century, scientists had partially explained the reasons for the beneficial effects of shungite. It was discovered that this mineral mainly consists of carbon, a significant part of which is in the form of spherical fullerene structures. Finally, the discovery of fullerene in shungite rocks gave a new impetus to shungite research [8].

The structure of shungite is an allotropic form of metastable carbon at the pre-graphite stage of coalification. In addition to carbon, the composition of shungite mined from the Zazhoginsky deposit in Karelia includes SiO_2_ (57.0 wt%), TiO_2_ (0.2 wt%), Al_2_O_3_ (4.0 wt%), FeO (0.6 wt%), Fe_2_O_3_ (1.49 wt%), MgO (1.2 wt%), MnO (0.15 wt%), K_2_O (1.5 wt%), and S (1.2 wt%). The product obtained by the thermal roasting of shungite (shungizite) at 1200–1400 °C contains small amounts of V (0.015 wt%), B (0.004 wt%), Ni (0.0085 wt%), Mo (0.0031 wt %), Cu (0.0037 wt%), Zn (0.0067 wt%), Co (0.00014 wt%) As (0.00035 wt%), Cr (0.0072 wt%), and other elements.

Shungite density is 2.1–2.4 g/cm^3^; porosity—max. 5%; compressive strength—100–120 MPa; electrical conductivity coefficient—1500 S/m; thermal conductivity coefficient—3.8 W/(m⋅K); and adsorption capacity—max. 20 m^2^/g.

Shungites differ in the composition of the mineral base (aluminosilicate, siliceous, carbonate) and the amount of shungite carbon. Shungite rocks with a silicate mineral base are subdivided into low-carbon shungite-bearing rocks (max. 5 wt% of C), medium-carbon shungites (5–25 wt% of C), and high-carbon shungites (25–80 wt% of C) [9].

Compounds with various chemical structures and properties can exhibit antioxidant activity. There are several classifications of antioxidants. First, they are divided into two groups: antioxidant enzymes (for example, superoxide dismutase, catalase, glutathioperoxidase, etc.) and low-molecular-weight non-enzymatic antioxidants (glutathione, ascorbic acid, flavonoids, etc.) [10]. In addition, it is possible to classify antioxidants according to their mechanism of action. Thus, there are antioxidants which are proton donors, traps for free radicals, or form chelate complexes with redox-active metal ions [11]. Typically, much of the recently published research evaluating the health benefits of exogenous antioxidants has focused on plant-derived compounds. This is primarily due to the fact that, despite the controversy about the effectiveness of antioxidants in the treatment of various diseases [12], numerous studies have proven not only the antioxidant properties of plant metabolites, but also their antibacterial, antiviral, cytotoxic, anticarcinogenic effects [13]. It should be noted that it is hard to find studies on the antioxidant properties of natural minerals and mineraloids in the literature. There are only a few data from studies on the antioxidant properties of zeolite [14,15].

This paper focuses on studying the antioxidant and cytotoxic activity of shungite, and determining the adsorption properties of shungite of different fractions.

## 2. Materials and Methods

### 2.1. Objects of Research

The objects of research were samples of shungite mined from deposits in Karelia (Karelskiy Shungitovyy Zavod, Petrozavodsk, Russia). Shungite was ground using a Pulverisette laboratory mill (DV-Ekspert, St. Petersburg, Russia), and 5 samples of different fractions were used: (1) Sh5 (5 µm); (2) Sh20 (20 µm); (3) ShT 20 (20 µm heat-treated); (4) Sh209 (209 µm); (5) ShT209 (209 µm heat-treated). Heat treatment was carried out in a vacuum for 2 h at a temperature of 900 °C.

The HEK293 cell line derived from human embryonic kidney cells was another object of research. The cell line was purchased from the Pokrovsky Stem Cell Bank (St. Petersburg, Russia).

The objects of research for the adsorption properties were mycotoxins: Aflatoxin B1, Ochratoxin, T-2 toxin, Deoxynivalenol (DON), Zearalenone, and Fumonisin. Mycotoxins were purchased from Moskhimtorg, Moscow, Russia.

### 2.2. Extraction of Shungite Water-Soluble Fraction

A weighed portion of shungite (1.00 g) was placed in a glass flask, and 10 mL of a 0.0022 M aqueous solution of orthophosphoric acid was added. A solution of orthophosphoric acid was chosen as an extractant since it is an eluent for determining the antioxidant activity on a Tsvet-Yauza-01-AA analyzer (Khimavtomatika, Moscow, Russia), and it is known that better extraction of lanthanides occurs in an acidified aqueous medium [16].

The resulting mixtures were thoroughly mixed for 1 h using a laboratory magnetic stirrer and kept in a closed container for three days at room temperature (this time is sufficient for the water-soluble fractions to be almost completely transferred from shungite to water) [17]. 

Then, the mixtures were centrifuged at 4500× *g* for 30 min in an Armed LC-04V laboratory centrifuge (Medmart, Moscow, Russia), and the supernatant was used to determine the antioxidant activity by amperometric and spectrophotometric methods. 

### 2.3. Determination of Antioxidant Activity by Amperometric Method

The antioxidant activity of the extracts was determined using a Tsvet-Yauza-01-AA analyzer (Khimavtomatika, Moscow, Russia) according to the measurement procedure (MP) for the content of antioxidants in drinks and food products, biologically active additives, and extracts of medicinal plants by the amperometric method, developed by Khimavtomatika (2007). The amperometric method for measuring the mass concentration of antioxidants is based on measuring the strength of the electric current arising from the oxidation of antioxidant molecules on the surface of the working electrode at a specific potential, which, after amplification, is converted into a digital signal. 

### 2.4. Determination of Antioxidant Activity by Spectrophotometric Method (DPPH Method) 

The method is based on the molecule binding ability of the reactive radical 2,2-diphenyl-1-picrylhydrazyl (DPPH) with antioxidants contained in the studied samples. Each extract was mixed with 2.85 mL of a freshly prepared 0.1 mM solution of 2,2-diphenyl-1-picrylhydrazyl (DPPH) in ethanol. The sample was incubated for 30 min at room temperature in the dark. The decrease in optical density at 515 nm (UV-3600, Shimadzu, Kyoto, Japan) was measured spectrophotometrically [18].

Solutions of precisely known concentration of ascorbic acid, trolox (a water-soluble analog of vitamin E), and quercetin were used as standards for the determination of antioxidant activity by the methods described above. 

Sample preparation included the preparation of a saturated solution, filtration, and mass measurement. Antioxidant (AO) activity was studied by a spectrophotometric method based on the inhibition of the stable chromogen radical 2,2-diphenyl-1-picrylhydrazyl (DPPH). In the visible region of the spectrum, DPPH in organic solvents has a broad absorption maximum at wavelengths of 515÷520 nm, which disappears when the radical interacts with suitable substances—donors of hydrogen atoms or free radicals of a different structure.

The reaction of DPPH with antiradical antioxidants (${\mathrm{DPPH}}^{•}$) occurs according to a serial–parallel mechanism. At the first stage (the rate-limiting stage of the reaction), the antioxidant molecule gives the radical the most mobile hydrogen atom:

(1) $${\mathrm{DPPH}}^{•}+\mathrm{AH}\to \mathrm{DPPH}-H+A^{•}.$$

At the second stage, the antioxidant radical ($A^{•}$) formed in reaction (1) attacks a new DPPH molecule in the para-position of the phenyl substituent:

(2) $$A^{•}+{\mathrm{DPPH}}^{•}\to A-\mathrm{DPPH}.$$

This also results in an uncolored reaction product. Reaction (1) can proceed by two independent mechanisms. The first, based on the direct abstraction of a hydrogen atom from an AO molecule, proceeds at the highest rate in non-polar solvents. The second, based on the transfer of an electron by an ionized phenolic AO molecule to a DPPH molecule, prevails in solvents with a high affinity for the proton.

### 2.5. Determination of the Cytotoxicity of Shungite

The Alamar Blue method was used to study cytotoxicity. This method is based on the conversion of a compound called Alamar Blue (resazurin) into a compound of resorufin along with living cells. Resazurin is a redox-blue dye that freely passes through the cell membrane to enter the cell, where it is reduced and converted to the fluorescent pink compound resorufin. Dead cells cannot reduce resazurin, and thus generate a fluorescent signal due to a loss of metabolic activity. The received signal was detected using a Qubit 3.0 fluorimeter (Tekhnokom, Novosibirsk, Russia); the intensity increased with an increase in the number of viable cells. To implement this method, 5000 HEK293 cells were inoculated into a 96-well Thermo Fisher plate (Thermo Fisher, Moscow, Russia) in 100 µL of full media. After 24 h, the analyzed preparation was added and incubated for 24–48 h at 37 °C; then 10 µL of Alamar Blue was added before incubating for a further 20 min.

The fluorescence intensity (excitation 560 ± 10 nm, emission 590 ± 10 nm) was measured on a Thermo Fisher plate reader (Thermo Fisher, Moscow, Russia). Each determination was carried out in triplicate. 

### 2.6. Determination of the Shungite Adsorption Value

A total of 50 cm^3^ of an aqueous solution of hydrochloric acid (with pH = 3.5), simulating gastric juice was poured into 10 Erlenmeyer flasks. The level of liquid was marked with a marker. Then, solutions of mycotoxin standards in 70% methanol were added to the flasks (5 flasks—T-2 toxin flasks, and in 5 others—Zearalenone) in the amount necessary to create a concentration gradient at the following levels: 50 μg/kg for Aflatoxin B1, 300 μg/kg for Ochratoxin, 100 μg/kg for T-2 toxin, 1000 μg/kg for DON, 1000 μg/kg for Zearalenone, and 2000 μg/kg for Fumonisin. Then, all the flasks were closed with stoppers and placed on an RK-2D shaker (Tochnye pribory, Moscow, Russia) for 10 min. (The working solution of mycotoxin is prepared in advance by dissolving the mycotoxin standard in 70% methanol.) After the specified time, 1 cm^3^ of the solution was sampled from each flask with an autosampler, and the mycotoxin content was measured—the actual value of the initial concentration of mycotoxins was obtained (control).

Portions of shungite samples weighing 5 g (a deliberate excess) were quantitatively introduced into the flasks with mycotoxins and placed on an RK-2D shaker heated to 37 °C (Tochnye pribory, Moscow, Russia) for 1 h. This method was used to simulate the time and conditions of the gastric residence of the shungite sample. Then, the supernatant liquid was separated from the sediment by decantation and examined for the content of free (unbound by the sorbent) mycotoxin by enzyme-linked immunosorbent assay (ELISA). Based on this value, the maximum adsorption value was calculated—the amount of toxin bound by the sorbent (in pure form, without dilution), during the time the shungite was in the stomach, equal to the difference between the initial amount of the added toxin and the amount of toxin in the supernatant liquid, separated after the incubation of the sample in an acidic solution and expressed in %.

All reagents were purchased from Moskhimtorg, Moscow, Russia.

### 2.7. Determination of the Shungite Desorption Value

A buffer solution simulating intestinal juice (phosphate-buffered PBS with pH = 7.4) was added to the sediment of each sample up to the initial mark. The contents of the flasks were kept for 3 h at 37 °C with constant stirring. This method was used to simulate the time and pH of the medium of the shungite sample residence in the intestine. After settling by decantation, the supernatant liquid was separated from the sediment and examined for mycotoxin content by enzyme-linked immunosorbent assay (ELISA). The value of desorption (in % of the adsorbed amount)—the amount of toxin released by shungite during its presence in the intestine, equal to the amount of toxin in the supernatant after incubation in a weakly alkaline medium—was deduced. 

All reagents were purchased from Moskhimtorg, Moscow, Russia.

### 2.8. Net Efficiency Calculation

Net efficiency was calculated according to the formula:

NE **=** Adsorption (% of control)—Desorption (% of adsorption), (3)

where NE—net efficiency, %; Adsorption—the amount of toxin associated with shungite during the gastric residence, %; Desorption—the amount of toxin released by shungite during intestine residence, %.

### 2.9. Statistical Analysis

All experimentations were achieved in triplicate, and results were given as a mean ± standard deviation. The data were subjected to the analysis of variance (ANOVA). The differences in the extracts were investigated by using a post hoc test (*p* < 0.05), and this test was performed in Statistica 10.0 (StatSoft Inc., 2007, Tulsa, OK, USA). 

## 3. Results

The results of determining the antioxidant activity of shungite extracts by the amperometric method are presented in Table 1.

The results of determining the antioxidant activity by the DPPH method are presented in Table 2.

**Figure 1:**
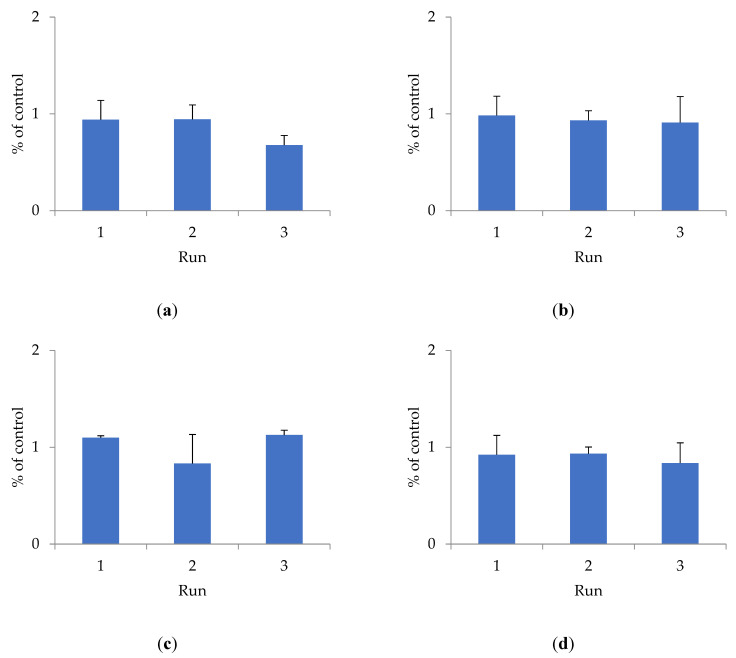

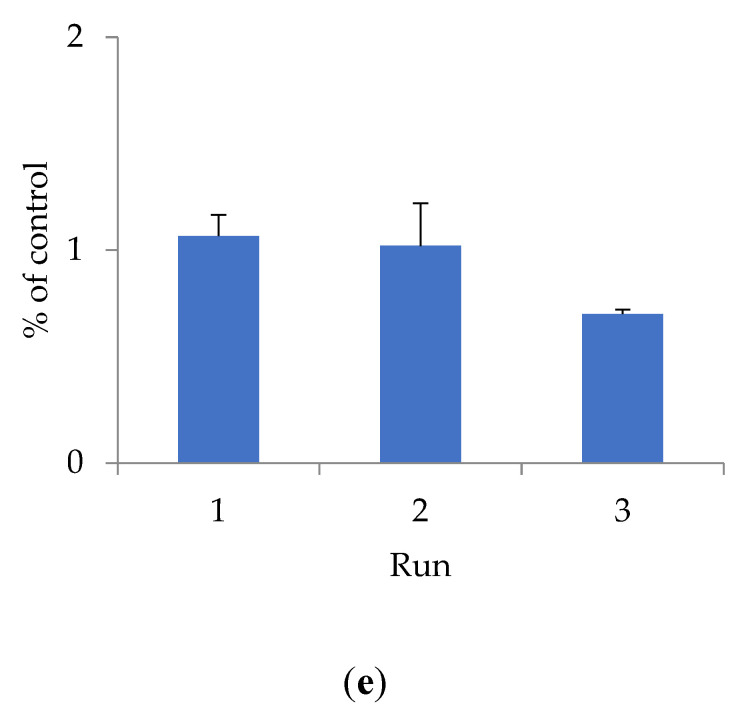
The effect of shungite on the HEK293 cell line, assessed by the Alamar Blue method: (**a**) Sh5; (**b**) Sh20; (**c**) ShT20; (**d**) Sh209; (**e**) ShT209. Data presented as a mean ± SD (*n* = 3).

To determine cytotoxicity, the effect of shungite samples of different fractions on the HEK293 cell line was studied using the Alamar Blue method (Figure 1).

The results of experiments on the sorption and desorption of mycotoxins for samples Sh209, ShT209, Sh20, Sh5, and ShT20 are presented in Table 3.

## 4. Discussion

The Sh20 sample had the maximal antioxidant activity as determined by the amperometric method—1.30 mg of ascorbic acid equivalents/g of shungite; 3.46 mg of trolox equivalents/g of shungite; and 0.99 mg of quercetin equivalents/g of shungite. Thus, the obtained data indicate that shungite has antioxidant properties, but these are about 1000 times less pronounced than those of quercetin.

The ShT20 sample was distinguished by the highest antioxidant activity (Table 2), determined by the DPPH method. However, when using this method, the differences between the samples were less pronounced, presumably because the molecules of the reactive radical 2,2-diphenyl-1-picrylhydrazyl bind with the same ease to all shungite samples and show less pronounced differences in the antioxidant activity between them. When using the amperometry method, the differences between the samples are more significant.

Our study showed that shungite samples had antioxidant properties, which were manifested in the ability to reduce oxidized components and to bind to free radicals (for example, the 2,2-diphenyl-1-picrylhydrazyl radical). According to this indicator, shungite Sh20 is superior to Sh209, ShT209 and Sh5, as well as common antioxidant sorbents of sedimentary zeolites (0.71 mg of quercetin equivalent/g), volcanic zeolites (0.62 mg of quercetin equivalent/g), and synthetic zeolites (0.42 mg of quercetin equivalent/g) [19]. The maximum antiradical activity was found in the ShT20 sample and amounted to 1.63 mg/g, 2.19 mg/g, and 1.11 mg/g when ascorbic acid, troloxa, and quercetin were used as standards, respectively. This indicator for shungite is about 4 times lower than that of sedimentary zeolite (8.93 mg of trolox equivalent/g) [20] and 2–3 times lower than that of volcanic zeolite (4.4–6.5 mg trolox equivalent/g) [21]. 

ShT20 possesses the highest adsorption capacity because its net efficiency (NE) value is the highest. In particular, for Aflatoxin B1 it is 98.8%, for Ochratoxin—100%, for T-2 toxin—81%, Deoxynivalenol—84%, Zearalenone—100%, and Fumonisin—95%.

The antioxidant activity of shungite may be due to the presence of various components in its composition. Previously, it was shown that the composition of shungite includes carbon, including in the form of fullerenes [22], silicates, as well as trace elements Fe, Ti, V, Ni, Cu, and Zn, represented mainly by sulfides (pyrite, pyrrhotite, sphalerite), and oxides (rutile) [23]. The antioxidant properties of fullerenes have been established both in in vitro and in vivo experiments [24]. Lei et al. (2016) studied the antioxidant activity of water-soluble fullerene derivatives. The ability of these compounds to exhibit antiradical activity against the DPPH radical was shown [25]. Gharbi et al. (2005) proved the protective effect of C60-fullerene, due to its pronounced antioxidant properties, in studies on rats with acute intoxication caused by carbon tetrachloride [26]. The optimal fraction of shungite exhibiting maximum antioxidant activity is proven to be 20 µm. Additional activity is conferred to shungite by heat treatment, which increases the extraction of active substances that bind free radicals and have a toxic effect on the HEK293 cell line. The shungite fraction ShT20 has the highest cytotoxicity. The obtained data are confirmed by other research [24,25,26].

In [27], it was shown that antitumor drugs could be designed on the basis of endometallofullerenes. Thus, polyhydroxylated endohedral fullerene CdC_82_(OH)_22_ forms particles about 22 nm in size in a physiological solution. Such particles at a dose of 10–7 mol/kg exhibit high antineoplastic activity in mice. The particles, showing practically no toxic effect in vitro and in vivo, inhibited the growth of tumors, interfering with the processes of tumor invasion into normal muscle tissue, and were detected in the tumor at a concentration of 0.05% of the administered dose. This distinguishes them from conventional antitumor drugs, the action of which is associated with a cytotoxic effect. According to the authors, these results indicate that fullerene derivatives, with an appropriately modified surface, have the potential to realize the dream of oncologists and chemotherapists—to create a highly effective, low-toxic, possibly universal antitumor drug [27].

## 5. Conclusions

This study established that shungite samples exhibit antioxidant activity, which manifests itself in the ability to reduce oxidized components and to bind to free radicals. The Sh20 sample had the maximal antioxidant activity as determined by the amperometric method—1.30 mg of ascorbic acid equivalents/g of shungite; 3.46 mg of trolox equivalents/g of shungite; and 0.99 mg of quercetin equivalents/g of shungite. The obtained data indicate that shungite has antioxidant properties, but these are about 1000 times less pronounced than those of quercetin. It was found that the ShT20 sample had a higher antioxidant activity, as determined by the DPPH method. Thus, it can be concluded that the optimal fraction of shungite exhibiting the maximum antioxidant activity is the fraction of 20 µm. According to the ability to reduce oxidized components and bind to free radicals, shungite Sh20 is superior to Sh209, ShT209, and Sh5, as well as common antioxidant sorbents of sedimentary zeolites (0.71 mg of quercetin equivalent/g), volcanic zeolites (0.62 mg of quercetin equivalent/g), and synthetic zeolites (0.42 mg of quercetin equivalent/g) [19]. The maximum antiradical activity was found in the ShT20 sample and amounted to 1.63 mg/g, 2.19 m/g, and 1.11 mg/g when ascorbic acid, trolox, and quercetin were used as standards, respectively. The ShT20 sample is distinguished by its adsorption properties because it is the most effective against all types of toxins.

Further studies, including the optimization of the antioxidant extraction conditions of shungite, and the analysis of the qualitative and quantitative composition of the obtained extracts, are required for a more accurate interpretation of the results. Depending on the chemical nature of the extractant, the obtained extracts differ significantly in the quantitative content of elements, which is essential for organizing the production of concentrates enriched with one or another set of the latter, considering the mineral content in the original shungite rock. Generalization of the data allows the statement that the mineral substance of shungite has a complex mineralogical and chemical composition, which is undoubtedly due to the nature of the initial biological material that participated in the formation of the organic and mineral matter of shungite and has a close genetic relationship with it. Individual elements are linked into complicated organometallic complexes, the presence of which in shungite was confirmed by studying its extraction products with organic solvents of different polarity by FTIR and UV/VIS spectroscopy.

Our study allows us to determine the future of shungite use. Shungite can find wide practical application in many branches of science and industry; in particular, shungite can be used as an alternative to activated carbon—a natural mineral absorbent for water purification (as a filter material). The microelements and biologically active components present in shungite determine its sorption, antioxidant properties, and properties that increase the extraction of active substances that bind free radicals and have a toxic effect on cell lines. The above properties make shungite an attractive material for agriculture, the feed industry, and poultry farming, since it can be used as a feed additive or fertilizer. In addition, due to its antioxidant properties, it can be used as an agent for preserving vegetables and treating animals [28]. Shungite is also irreplaceable in the food, pharmaceutical, and cosmetic industries [29].

High dispersion and porosity, which are responsible for the sorption capacity of the shungite substance, provide the chemical resistance of rocks in aggressive environments [30]. Therefore, it is possible to discover new structural forms of carbon within, and useful properties of, shungite substance, which will make it possible to create effective technologies for the practical use of shungite rocks, particularly in the production of fullerenes and other carbon nanoclusters in the chemical industry [31].

## Figures and Tables

**Table 1:** Antioxidant activity (mg/g according to the standard) of shungite extracts (amperometric method).

Samples	Standard		
	I	II	III
Sh5	0.65 ± 0.05	1.73 ± 0.12	0.49 ± 0.03
Sh20	1.30 ± 0.08 *	3.46 ± 0.20 *	0.99 ± 0.06 *
ShT20	0.86 ± 0.03 *	2.29 ± 0.08 *	0.65 ± 0.02 *
ShT209	0.62 ± 0.01	1.65 ± 0.03	0.47 ± 0.01
ShT209	0.71 ± 0.01	1.88 ± 0.04	0.53 ± 0.01

Sh5 (size fraction 5 µm); Sh20 (size fraction 20 µm); ShT 20 (size fraction 20 µm, heat treated); Sh209 (size fraction 209 µm); ShT209 (size fraction 209 µm, heat treated). I—ascorbic acid; II—trolox; III—quercetin. Data presented as a mean ± SD (*n* = 3). Values in columns followed by the symbol “*” do differ significantly (*p* < 0.05), as assessed by the post hoc test (Tukey test).

**Table 2:** Antioxidant activity (mg/g according to the standard) of shungite extracts (DPPH method).

Samples	Standard		
	I	II	III
Sh5	1.36 ± 0.12	1.52 ± 0.09	0.79 ± 0.04
Sh20	1.34 ± 0.14	1.50 ± 0.06	0.77 ± 0.03
ShT20	1.63 ± 0.12	2.19 ± 0.12 *	1.11 ± 0.04 *
ShT209	0.85 ± 0.06 *	1.14 ± 0.08 *	1.05 ± 0.06
ShT209	1.08 ± 0.08	1.43 ± 0.06	1.06 ± 0.02

Sh5 (size fraction 5 µm); Sh20 (size fraction 20 µm); ShT 20 (size fraction 20 µm, heat treated); Sh209 (size fraction 209 µm); ShT209 (size fraction 209 µm, heat treated). I—ascorbic acid; II—trolox; III—quercetin. Data presented as a mean ± SD (*n* = 3). Values in columns followed by the symbol “*” do differ significantly (*p* < 0.05), as assessed by the post hoc test (Tukey test).

**Table 3:** Data on sorption and desorption of mycotoxins.

Mycotoxin	Initial Concentration, μg/kg	Adsorption, %					Desorption, %					N.E., %				
		1	2	3	4	5	1	2	3	4	5	1	2	3	4	5
Aflatoxin B1	50	95	94	100	96	97.0	5.0	8.0	1.2	12.0	8.0	90.0	86.0	98.8	84.0	89.0
Ochratoxin	300	100	100	100	100	100.0	0.0	0.0	0.0	0.0	0.0	100.0	100.0	100.0	100.0	100.0
T-2 toxin	100	96	94	93	93	90.0	10.0	12.0	13.0	13.0	13.0	86.0	82.0	81.0	81.0	77.0
Deoxynivalenol	1000	98	93	96	96	98.0	9.0	9.0	12.0	12.0	14.0	89.0	84.0	84.0	84.0	84.0
Zearalenone	1000	100	100	100	100	100.0	0.0	0.0	0.0	0.0	0.0	100.0	100.0	100.0	100.0	100.0
Fumonisin	2000	92	97	100	98	98.0	4.6	5.3	4.8	4.6	4.4	87.4	91.7	95.0	93.4	93.6

Samples 1—Sh5, 2—Sh20, 3—ShT20, 4—Sh209, 5—ShT209. Data presented as a mean S.D. (*n* = 3).

## Data Availability

Data are contained within the article.
